# DASH DIET WITH CULTURALLY TAILORED RECIPES: A PSYCHOEDUCATIONAL SESSION FOR BLACK WOMEN WITH HYPERTENSION

**DOI:** 10.1093/geroni/igae098.0455

**Published:** 2024-12-31

**Authors:** Angela Groves, Kate Hamann, Maria Roche-Dean

**Affiliations:** Western Michigan University, Kalamazoo, Michigan, United States; Ascension Borgess Medical Hospital, Kalamazoo, Michigan, United States; University of Kansas, Kansas City, Kansas, United States

## Abstract

The prevalence of hypertension among non-Hispanic Black women in the United States is alarming because they develop hypertension at an earlier age and have higher blood pressures when compared to White women. Approximately 55.3% of Black women have hypertension, which puts them at significant risk of developing heart disease, kidney disease, and cerebral vascular accidents. The Dietary Approaches to Stop Hypertension (DASH) diet, when combined with lower sodium intake, has been associated with a reduction in systolic blood pressure by 10 mm Hg in a four-week intervention study that included 50% African American women with pre-hypertension or stage 1 hypertension. Although, the DASH diet does not consider cultural food preferences. Studies among Black adults have shown that when the DASH diet incorporated culturally traditional foods, adherence to the DASH diet improved, which lowered blood pressure. A key factor in understanding the health behavior of Black women is their general knowledge of hypertension and low-sodium diets. This psychoeducation session was part of an 8-week peer (dyadic) support mixed methods study that included the DASH diet and culturally tailored recipes to improve diet adherence and reduce systolic blood pressure. Purposive sampling was used to secure a sample of 34 older Black women from local churches and one senior center to participate in a one-hour DASH diet education session. Psychoeducation was reinforced with weekly newsletters and opportunities for one-on-one discussions via email or phone. Study results indicated that participants were able to successfully reduce systolic pressure. We discuss the implications for hypertension management.

